# Detection of Influenza C Virus Infection among Hospitalized Patients, Cameroon

**DOI:** 10.3201/eid2503.181213

**Published:** 2019-03

**Authors:** Richard Njouom, Gwladys Chavely Monamele, Burcu Ermetal, Serge Tchatchouang, Sylvie Moyo-Tetang, John W. McCauley, Rodney S. Daniels

**Affiliations:** Centre Pasteur du Cameroon, Yaounde, Cameroon (R. Njouom, G.C. Monamele, S. Tchatchouang);; The Francis Crick Institute, London, United Kingdom (B. Ermetal, J.W. McCauley, R.S. Daniels);; Centre Hospitalier d’Essos, Yaounde (S. Moyo-Tetang)

**Keywords:** Influenza C, viruses, hospitalized patients, Cameroon, virus, influenza

## Abstract

We report 3 cases of influenza C virus in children hospitalized with severe acute respiratory infection in Cameroon. Two of these case-patients had grave clinical manifestations, but all 3 recovered. The lack of specific antiviral drugs for influenza C virus highlights the need to identify and describe cases involving this virus.

Four types of influenza viruses are known: A, B, C, and D (*1*). Unlike influenza viruses types A and B, influenza C viruses generally cause a mild respiratory illness (*2*). However, some cases of lower respiratory infections have been described in children (*1**–**3*). In recent years, severe illness due to influenza C virus has been reported from different geographic regions, but few data have come from Africa (*3**–**6*). Previous detection of influenza C virus in Cameroon reported 2 cases among 561 patients with influenza-like illness (*7*). We identified 3 cases of influenza C virus infection among hospitalized patients with severe acute respiratory infection (SARI) in Cameroon.

Respiratory samples were collected as part of the influenza surveillance system in Centre Hospitalier d’Essos, a SARI site located in the central region of Cameroon that has been involved in surveillance activity since the onset of the program in 2007. At this site, hospitalized SARI patients are screened for other respiratory pathogens, including bacteria and viruses, within the framework of an internal project running concomitantly with influenza surveillance activity since January 2017. The Cameroon National Ethics Committee gave ethics clearance for this study (document no. 2017/03/876/CE/CNERSH/SP). Each child’s parent or guardian also provided written consent before sample collection. While awaiting laboratory analyses, the children received presumptive treatments, including antipyretic, antimicrobial, and antimalarial drugs. No antiviral drugs are available to treat patients with influenza C virus infection.

Nasopharyngeal swab specimens were collected in universal transport medium and transported to Centre Pasteur du Cameroon (Yaounde, Cameroon), where we performed analyses. We extracted RNA from the samples using a QIAamp Viral RNA Mini Kit (QIAGEN, http://www.qiagen.com) according to the manufacturer’s instructions. We then analyzed samples for the presence of 33 respiratory pathogens using a real-time reverse transcription PCR (Fast-Track Respiratory Pathogens 33 PCR kit; Fast-Track Diagnostics Ltd., https://www.fast-trackdiagnostics.com), obtained through the International Reagent Resource Program (https://www.internationalreagentresource.org). We ran the assay in an Applied Biosystems Prism 7500 thermocycler (Thermo Fisher Scientific, Inc., https://www.thermofisher.com) and considered all reactions with cycle thresholds <37 positive for the tested pathogens. We confirmed influenza C virus in samples from 3 children, 11 months, 3 years, and 4 years of age. The age distribution of the patients is compatible with reports showing that most humans acquire antibodies to influenza C virus early in life (*3*). 

Other studies have shown that influenza C infection in infants can be severe enough to require hospitalization, compared with infection in adults (*3**,**8*). All 3 patients had fever, cough, rhinorrhea, asthenia, and conjunctivitis, but 2 had additional grave clinical manifestations. One patient had exacerbated symptoms, including dyspnea, vomiting, diarrhea, and stage II coma; another had an altered general state. All 3 patients recovered. 

Unlike similar studies in which influenza C was identified mostly in patients with co-infections of other respiratory pathogens (*4**,**5*), we did not detect co-infection in these 3 patients. Further, absence of underlying or preexisting medical conditions in the children indicates that their illnesses were solely caused by influenza C virus infection. Because all 3 cases were detected in the course of a project focusing on hospitalized patients in a single center, additional cases of influenza C infection among outpatients and hospitalized patients are likely.

We used influenza C sequences available in the GISAID EpiFlu database (https://www.gisaid.org) as of April 18, 2018, to design primers for whole-genome sequencing by next-generation sequencing and Sanger sequencing of the hemagglutinin-esterase (HE) gene. Based on the HE gene, 6 distinct clades of influenza C virus have been identified: C/Taylor/1233/47, C/Mississippi/80, C/Aichi/1/81, C/Yamagata/26/81, C/Kanagawa/1/76, and C/Sao Paulo/378/82 (*5*). Two of the patients’ specimens yielded good sequence data, and maximum-likelihood HE gene phylogenetic analysis showed the Cameroon viruses cluster in the C/Sao Paolo/378/82 lineage (Figure), a dominant clade that has been detected in several other countries (*5**,**9**,**10*).

**Figure:**
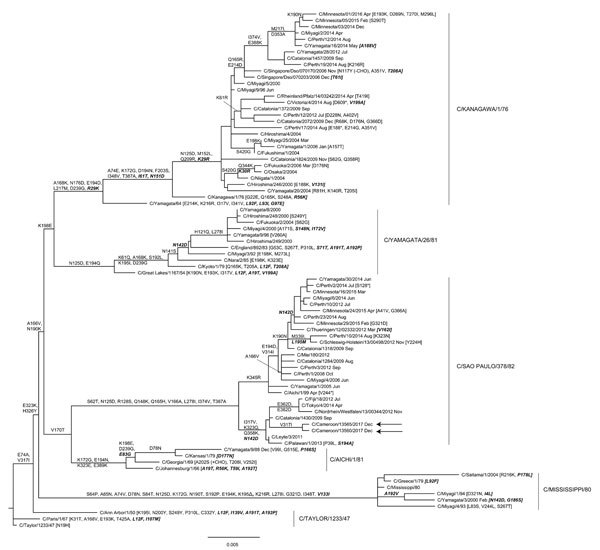
Hemagglutinin-esterase gene phylogeny for influenza C viruses detected in Cameroon compared with reference viruses. The phylogeny is based on 80 full-length open-reading frames downloaded from the GISAID EpiFlu database (https://www.gisaid.org), with signal peptide coding regions and stop codons removed, yielding products of 1,923 nt. The phylogeny was estimated by using RaxML version 8.2.X (https://sco.h-its.org/exelixis/software.html) with a general time-reversible plus gamma substitution model and then annotated with amino acid substitutions defining nodes and individual virus gene products by using treesub (https://github.com/tamuri/treesub/blob/master/README.md). The phylogeny was visualized by using FigTree version 1.4.4 (http://tree.bio.ed.ac.uk/software/figtree). Sequences for the 2 viruses from Cameroon (arrows) have been deposited in the EpiFlu database under accession nos. EPI1259829 (C/Cameroon/13560/2017) and EPI1259835 (C/Cameroon/13565/2017). Scale bar indicates nucleotide substitutions per site.

Studies have shown variability in the circulation of influenza C virus, with peaks in winter and spring seasons (*3**,**4*). These 3 cases all occurred in December 2017, possibly indicating seasonality of influenza C infection in Cameroon, but identification of more cases is required to confirm this hypothesis.

The recent identification of severe illnesses related to influenza C virus infection in Cameroon and the lack of influenza C–specific antiviral drugs highlight the importance of integrating molecular testing for this virus into existing inpatient and outpatient sentinel surveillance systems and for in-depth studies of the epidemiology of influenza C viruses. This process could lead to predominant circulating strains of influenza C virus being included in seasonal influenza vaccines to protect vulnerable populations.

## References

[1] Centers for Disease Control and Prevention, National Center for Immunization and Respiratory Diseases (NCIRD). Types of influenza viruses. Influenza (Flu). 2017 Sep 27 [cited 2017 Dec 17]. https://www.cdc.gov/flu/about/viruses/types.htm

[2] Wang M, Veit M. Hemagglutinin-esterase-fusion (HEF) protein of influenza C virus. Protein Cell. 2016;7:28–45. 10.1007/s13238-015-0193-x 26215728 PMC4707155

[3] Matsuzaki Y, Katsushima N, Nagai Y, Shoji M, Itagaki T, Sakamoto M, et al. Clinical features of influenza C virus infection in children. J Infect Dis. 2006;193:1229–35. 10.1086/502973 16586359

[4] Thielen BK, Friedlander H, Bistodeau S, Shu B, Lynch B, Martin K, et al. Detection of influenza C viruses among outpatients and patients hospitalized for severe acute respiratory infection, Minnesota, 2013–2016. Clin Infect Dis. 2017;•••:23. 29069373 10.1093/cid/cix931 PMC5862734

[5] Jelley L, Levy A, Deng YM, Spirason N, Lang J, Buettner I, et al. Influenza C infections in Western Australia and Victoria from 2008 to 2014. Influenza Other Respi Viruses. 2016;10:455–61. 10.1111/irv.12402 27373693 PMC5059950

[6] Onyango CO, Njeru R, Kazungu S, Achilla R, Bulimo W, Welch SR, et al. Influenza surveillance among children with pneumonia admitted to a district hospital in coastal Kenya, 2007-2010. J Infect Dis. 2012;206(Suppl 1):S61–7. 10.1093/infdis/jis536 23169974 PMC3502370

[7] Njouom R, Yekwa EL, Cappy P, Vabret A, Boisier P, Rousset D. Viral etiology of influenza-like illnesses in Cameroon, January-December 2009. J Infect Dis. 2012;206(Suppl 1):S29–35. 10.1093/infdis/jis573 23169968 PMC7107314

[8] Calvo C, García-García ML, Centeno M, Pérez-Breña P, Casas I. Influenza C virus infection in children, Spain. Emerg Infect Dis. 2006;12:1621–2. 10.3201/eid1210.051170 17176596 PMC3290929

[9] Potdar VA, Hinge DD, Dakhave MR, Manchanda A, Jadhav N, Kulkarni PB, et al. Molecular detection and characterization of Influenza ‘C’ viruses from western India. Infect Genet Evol. 2017;54:466–77. 10.1016/j.meegid.2017.08.005 28803969

[10] Odagiri T, Matsuzaki Y, Okamoto M, Suzuki A, Saito M, Tamaki R, et al. Isolation and characterization of influenza C viruses in the Philippines and Japan. J Clin Microbiol. 2015;53:847–58. 10.1128/JCM.02628-14 25552361 PMC4390655

